# The hypoxia conditioned mesenchymal stem cells promote hepatocellular carcinoma progression through YAP mediated lipogenesis reprogramming

**DOI:** 10.1186/s13046-019-1219-7

**Published:** 2019-05-29

**Authors:** Yang Liu, Haozhen Ren, Yuan Zhou, Longcheng Shang, Yuheng Zhang, Faji Yang, Xiaolei Shi

**Affiliations:** Department of Hepatobiliary Surgery, The Affiliated Drum Tower Hospital of Nanjing University Medical School, NO.321 Zhongshan Road, Nanjing, Jiangsu 210008 People’s Republic of China

**Keywords:** Hypoxia, Mesenchymal stem cells, YAP, Lipogenesis

## Abstract

**Background:**

Tumor microenvironment (TME) plays a very important role in cancer progression. The mesenchymal stem cells (MSC), a major compartment of TME, have been shown to promote hepatocellular carcinoma (HCC) progression and metastasis. As hypoxia is a common feature of TME, it is essential to investigate the effects of hypoxia on MSC during HCC progression.

**Methods:**

The effects of hypoxia on MSC mediated cell proliferation and HCC progression were measured by cell counting kit-8 (CCK-8) assay, Edu incorporation assay and xenograft model. The role of cyclooxygenase 2 (COX2) during this process was evaluated via lentivirus mediated COX2 knockdown in MSC. We also assessed the levels and localization of yes-associated protein (YAP) in HCC cells by immunofluorescence, western blot and real-time PCR, in order to detect the alterations of Hippo pathway. The changes in lipogenesis was examined by triacylglycerol (TG) levels, BODIPY staining of neutral lipid, and lipogenic enzyme levels. The alterations in AKT/mTOR/SREBP1 pathway were measured by western blot. In addition, to evaluate the role of prostaglandin E receptor 4 (EP4) in MSC mediated cell proliferation under hypoxia, we manipulated the levels of EP4 in HCC cells via small interfering RNA (siRNA), EP4 antagonist or agonist.

**Results:**

We found that MSC under hypoxia condition (hypo-MSC) could promote proliferation of HCC cell lines and tumor growth in xenograft model. Hypoxia increased COX2 expression in MSC and promoted the secretion of prostaglandin E_2_ (PGE_2_), which then activated YAP in HCC cells and led to increased cell proliferation. Meanwhile, YAP activation enhanced lipogenesis in HCC cell lines by upregulating AKT/mTOR/SREBP1 pathway. Knockdown or overexpression of YAP significantly decreased or increased lipogenesis. Finally, EP4 was found to mediate the effects of hypo-MSC on YAP activation and lipogenesis of HCC cells.

**Conclusions:**

Hypo-MSC can promote HCC progression by activating YAP and the YAP mediated lipogenesis through COX2/PGE_2_/EP4 axis. The communication between MSC and cancer cells may be a potential therapeutic target for inhibiting cancer growth.

**Electronic supplementary material:**

The online version of this article (10.1186/s13046-019-1219-7) contains supplementary material, which is available to authorized users.

## Background

Recently, the communication between tumor microenvironment (TME) and cancer cells has been increasingly appreciated as a pivotal contributor to tumor progression [1, 2]. One important component of TME is the mesenchymal stem cells (MSC), which are multipotent cells that reside in various tissues, and get recruited to primary tumor site from the bone marrow in response to tumor derived soluble factors [1, 3]. The link between MSC and tumor progression was established by the promoting effects from MSC on tumor growth and metastasis in mouse lymphoma, melanoma and breast cancer models when injected together with tumor cells. This effect is mainly mediated by the numerous factors and chemokines produced by MSC, such as transforming growth factor-β (TGFβ), CC-chemokine ligand 5 (CCL5), CXC-chemokine ligand 10 (CXCL10) and CXCL12 [1]. A characteristic phenomenon of TME in all solid tumor types, including hepatocellular carcinoma (HCC), is hypoxia, which has been found to promote angiogenesis, metabolic reprogramming, extracellular matrix remodeling and epithelial–mesenchymal transition (EMT) [4]. However, the role of MSC in HCC progression, especially under the hypoxia condition, has not been fully investigated.

Yes-associated protein (YAP) is the central transcriptional co-activator of Hippo pathway. It controls the trans-activation of a variety of target genes to regulate organ size, promote cell proliferation and inhibit apoptosis [5]. Recent studies confirmed the role of YAP in HCC progression, and found that YAP activity was increased during the early development of liver cancer [6, 7]. Moreover, liver-specific overexpression of YAP leads to hepatomegaly and subsequent tumor formation [8, 9].

Recently, altered lipid metabolism has been recognized as a hallmark of cancer [10]. Continuous de novo lipogenesis provides cancer cells with membrane building blocks, signaling lipid molecules and post-translational protein modifications to support rapid cell proliferation [11]. Increased lipid biosynthesis has been found to promote HCC development; moreover, the suppression of fatty acid synthase (FASN), a rate-limiting enzyme in lipogenesis, could impair the growth of human HCC cells [12]. Emerging evidences suggest that YAP can coordinate nutrient availability with cell growth and tissue homeostasis [13, 14]. Also, YAP was found to participate in metabolism regulation, such as glycolysis and lipogenesis [12, 13], in order to promote HCC progression. Therefore, the detailed mechanisms of how lipid metabolism is altered in HCC and how it is the related to YAP activation would be important questions to address.

In this study, we found that the MSC under hypoxia condition could promote HCC progression. More specifically, we found that hypoxia increased cyclooxygenase 2 (COX2) expression in MSC, leading to enhanced secretion of prostaglandin E_2_ (PGE_2_). Furthermore, the PGE_2_ secreted by MSC activated YAP in HCC cells, which then increased lipogenesis and promoted cell proliferation.

## Methods

### Cell lines and cell culture

HCC cell lines 7402 and Hep3b were purchased from the Shanghai Institutes for Biological Sciences. HCC cell line 7402 was cultured in RPMI 1640 medium (Corning Inc., Corning, NY, USA), and Hep3b was cultured in MEM medium (Corning Inc.). Both mediums were supplemented with 10% fetal calf serum (FBS), 100 U/ml penicillin and 100 g/ml streptomycin. Cells were maintained in a humidified incubator with 5% CO_2_ at 37 °C. Human umbilical MSC were purchased from ScienCell (San Diego, California, USA) and cultured in DMEM/F-12 medium (Corning Inc.). MSC at passage 3–10 were used for experiments. When MSC grew to about 80% confluence, they were switched to serum-free medium and cultured for 48 h at 37 °C under hypoxic (1.5% O_2_) (hypo-MSC) or normoxic (21.0% O_2_) conditions. The condition medium (CM) was harvested, purified by centrifugation and frozen at − 80 °C for further experiment.

### COX2 knockdown in MSC and YAP overexpression in HCC cell lines

To inhibit COX2 in MSC, lentivirus carrying shRNA against COX2 (GeneChem, Shanghai, China) was used to infect MSC [named MSC-COX2(−)]. The PGE_2_ levels in supernatant was detected by ELISA (enzyme-linked immunosorbent assay) kit (R&D System, Minneapolis, Minnesota, USA). To overexpress YAP in HCC cell lines, cells were infected with lentivirus carrying YAP gene (GeneChem). The sequences for the COX2 shRNA and YAP gene were listed in Additional file 1: Table S4.

### Reagents

PGE_2_, CAY10598 (EP4 agonist) and GW627368X (EP4 inhibitor) were bought from Cayman Chemical (Ann Arbor, MI, USA). AKT inhibitor, LY294002 was bought from Selleck Chemicals (Houston, TX, USA), SREBP1 inhibitor, Fatostatin was bought from MedChemExpress (MCE, Monmouth Junction, NJ, USA).

### Western blot and immunoprecipitation (IP)

Proteins were extracted from cells using RIPA buffer (KeyGEN BioTECH, Nanjing, China) with phenylmethylsulfonyl fluoride (PMSF) at 4 °C for 30 min. Western blot and IP were performed as previously described [15, 16]. Primary antibodies were listed in Additional file 1: Table S1.

### RNA isolation and quantitative real-time PCR (qRT-PCR)

Total RNA was isolated from HCC cells by TRIzol, followed by real-time PCR analysis with ABI 7500 (Applied Biosystems, Foster City, CA, USA). 18 s was used as an internal control. The 2^-ΔΔCT^ method was employed to determine the relative mRNA expression. The primer sequences were listed in Additional file 1: Table S2.

### Cell viability assay

Seven thousand four hundred two and Hep3b cells were plated in 96-well plates at the density of 2 × 10^3^/well and cultured overnight. On the next day, their medium was replaced with CM (100 μL/well) or fresh medium supplemented with PGE_2_. Cell viability was determined by cell counting kit-8 (CCK-8) assay (MCE).

### Small interfering RNA (siRNA) and transfection

siRNAs against YAP and prostaglandin E receptor 4 (EP4), as well as control siRNAs were obtained from RiboBio (GuangZhou, China). The detailed sequences of siRNAs were listed in Additional file 1: Table S3. Transfection with siRNAs and miRNAs were completed using riboFECT™ CP (RiboBio) according to the manufacturer’s instruction.

### Quantification of neutral lipid and triacylglycerol (TG)

The lipophilic fluorescence dye BODIPY 493/503 (Invitrogen, ThermoFisher Scientific, Eugene, OR, USA) was used to monitor the content of neutral lipids in HCC cells as previously described [17]. The TG in cells and tissues were measured by EnzyChrom™ Triglyceride Assay Kits (BioAssay Systems, Hayward, CA, USA) following manufacturer’s protocol.

### Immunofluorescence staining

HCC cells were seeded in 24-well cell culture cluster. After treatment, cells were fixed with 4% paraformaldehyde for 20 min, permeabilized with 0.5% Triton X-100 in PBS at room temperature for 15 min, and then blocked with 10% bovine serum albumin in PBS for 1 h. Subsequently, cells were incubated with primary antibodies against YAP (1:100, Abcam, Cambridge, MA, UK) or SREBP1(1:50, Santa Cruz Biotechnology, Dallas, TX, USA) at 4 °C overnight, followed by incubation with goat anti-rabbit or anti-mouse IgG H&L antibodies (Alexa Fluor® 488) (1200, Abcam) for 1 h. Nuclei were counterstained with 4′,6-diamidino-2-phenylindole (DAPI, KeyGEN BioTECH) and images were captured using fluorescence microscopy.

### Edu assay

Cells were seeded on glass coverslips in 24 well plates. After treatment, cells were incubated with Edu (Beyotime Biotechnology, Shanghai, China) for 2 h at 37 °C, and fixed in 4% paraformaldehyde. After permeabilization with 0.5% Triton-X, the cells were reacted with click additive solution (Beyotime Biotechnology) for 30 min. Subsequently, the DNA contents of the cells were stained with Hoechst 33342 for 10 min and visualized under a fluorescence microscope. Edu positive cells were quantified from three randomly selected fields in each well, and each experiment was repeated for three times.

### In vivo tumorigenesis assays

A total of 1 × 10^6^ tumor cells were injected alone or co-injected with MSC (2 × 10^5^) into 4-week-old BALB/c nude mice subcutaneously (*n* = 6 each group). After 4 weeks, mice were sacrificed, and the tumors were harvested, weighed, and saved for further studies. All procedures were approved by the Ethics Committee for Animal Experimentation of the Affiliated Drum Tower Hospital of Nanjing University Medical School. The harvested tumors were stained with Ki-67 (Abcam), YAP (Abcam) and CD90 (Abcam) by immunohistochemistry (IHC).

### Statistical analysis

All the data are expressed as mean ± SD. Student *t* test and one-way analysis of variance (ANOVA) were used to compare the data. *P* < 0.05 was considered statistically significant.

## Results

### Hypo-MSC promotes the proliferation of HCC cells both in vitro and in vivo

**Fig. 1:**
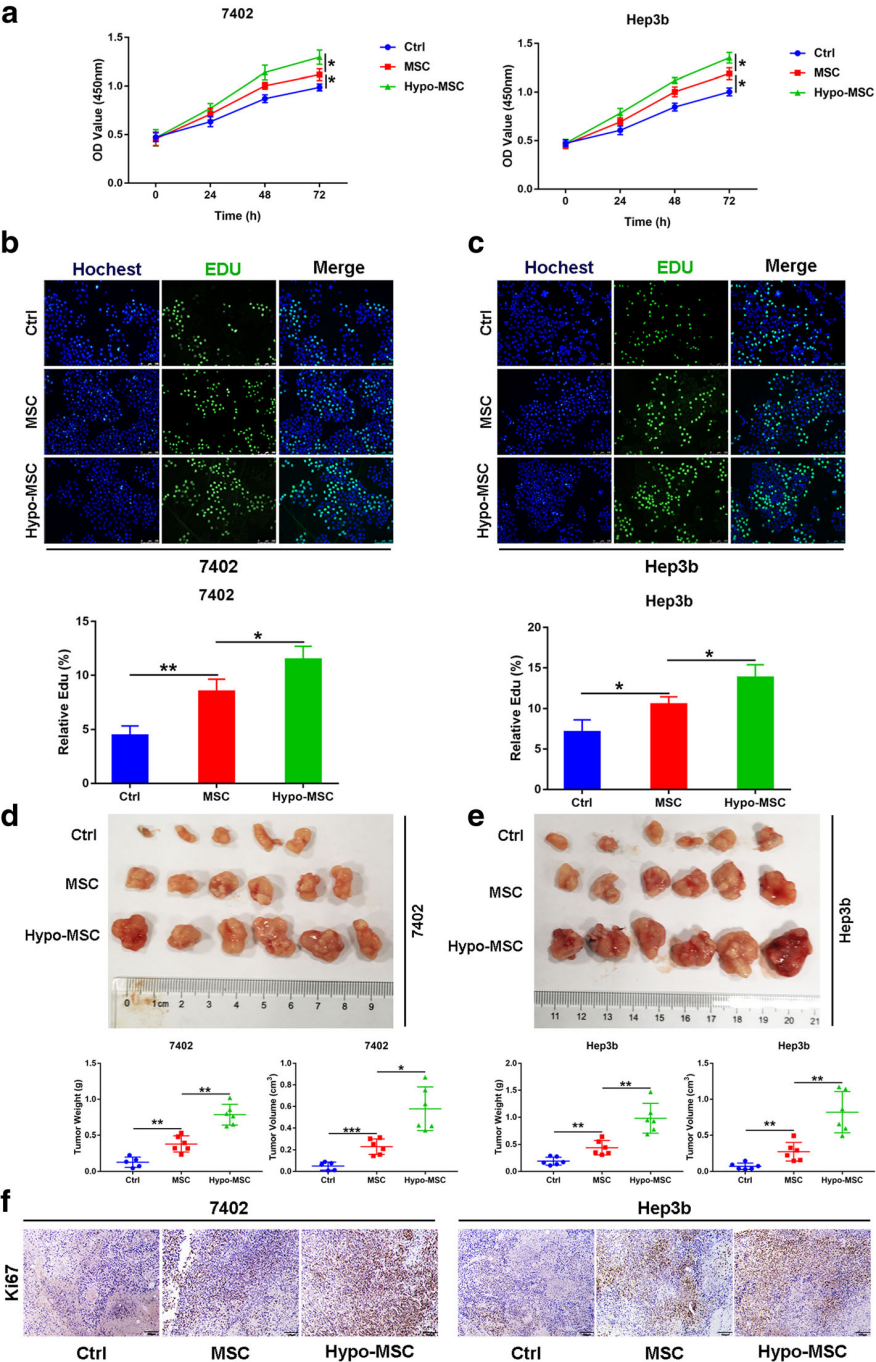
Hypo-MSC promotes the proliferation of HCC cells both in vitro and in vivo. **a** The proliferation ability of 7402 and Hep3b under indicated conditions (*n* = 3). **b-c** The representative images and quantification of Edu positive cells in 7402 and Hep3b under indicated conditions (*n* = 3). **d-e** Images of xenograft tumors, tumor volume and weight measurement of 7402 or Hep3b injected alone or co-injected with MSC or hypo-MSC (*n* = 5–6). **f** Representative images of Ki-67 in 7402 or Hep3b injected alone or co-injected with MSC or hypo-MSC. (*p < 0.05, **p < 0.01, ***p < 0.001, hypo-MSC: hypoxia conditioned MSC)

To explore the effects of MSC under hypoxia condition (hypo-MSC) on HCC cell proliferation, we performed CCK-8 assays and found that the CM of MSC under normoxia and hypoxia conditions could both promote HCC cells growth, but the hypo-MSC could increase cell growth more robustly (Fig. 1a). Consistently, Edu incorporation assay showed increased proliferation of HCC cells in the presence of either MSC or hypo-MSC-CM (Fig. 1b, c). Next, we tested whether hypo-MSC could promote HCC growth in vivo with xenograft model. Seven thousand four hundred two or Hep3b cells were subcutaneously implanted into nude mice, either alone or co-implanted with MSC or hypo-MSC. By measuring tumor mass and volume, we found that the tumors with hypo-MSC co-injection exhibited the fastest growth rate (Fig. 1d, e). Consistently, these tumors showed higher Ki-67 levels compared to other groups, as revealed by IHC staining (Fig. 1f and Additional file 2: Figure S1). Taken together, these findings demonstrated that hypo-MSC could promote HCC cell proliferation both in vitro and in vivo.

### Hypo-MSC promotes HCC progression through COX2/PGE_2_ axis

**Fig. 2:**
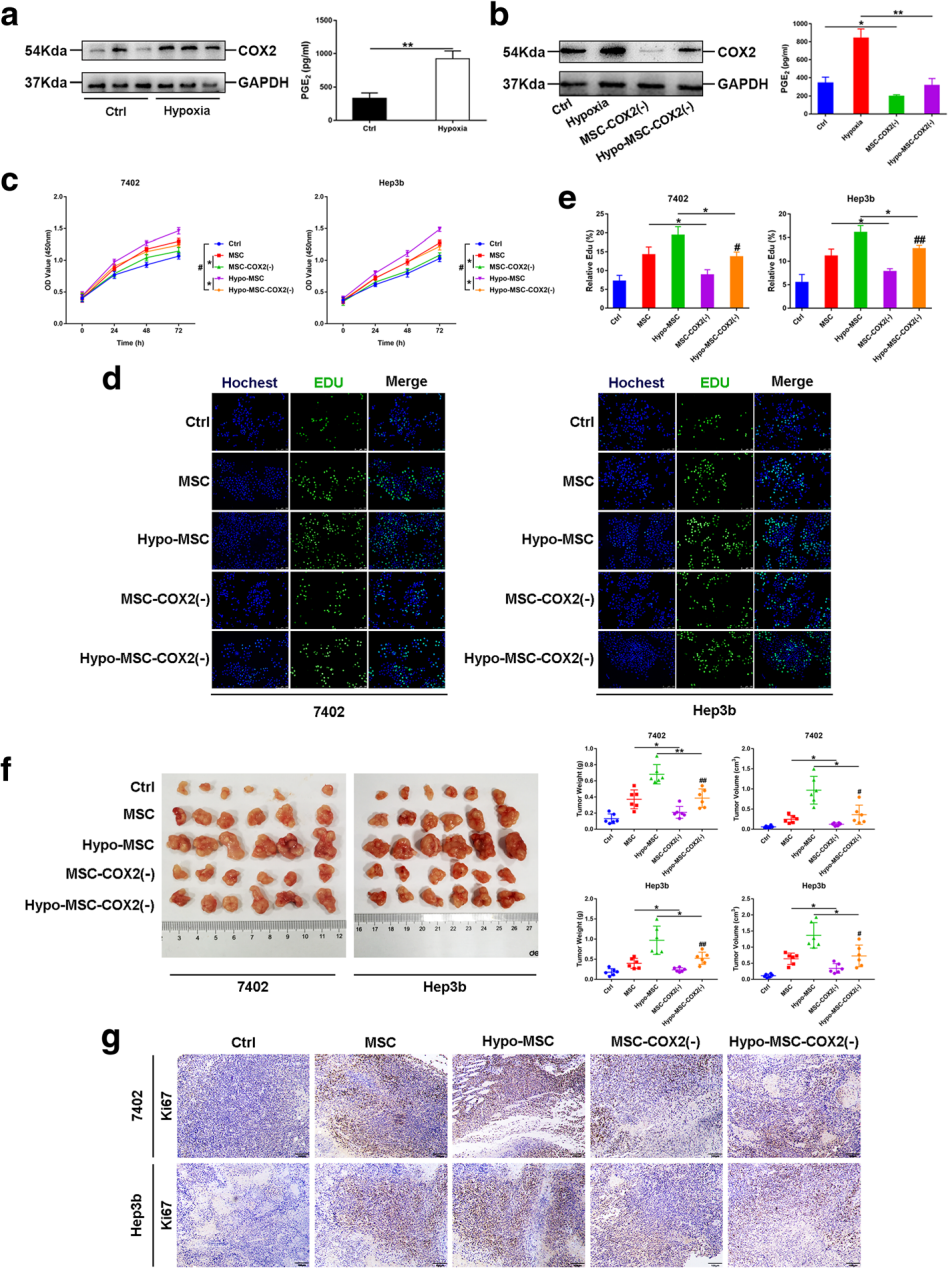
Hypo-MSC promotes HCC progression through COX2/PGE_2_ axis. **a** Protein levels of COX2 and PGE_2_ secreted by MSC under normal or hypoxia condition (*n* = 3). **b** Protein levels of COX2 and PGE_2_ secreted by MSC after COX2 knockdown by lentivirus under normal or hypoxia condition (*n* = 3). **c** The proliferation ability of 7402 and Hep3b under indicated conditions (*n* = 3). **d-e** Representative images and quantification of Edu positive cells in 7402 and Hep3b under indicated conditions (*n* = 3). **f** Images of xenograft tumors, tumor volume and weight measurement of 7402 or Hep3b injected alone or co-injected with MSC (*n* = 6). **g** The representative images of Ki-67 of xenograft tumors in 7402 or Hep3b injected alone or co-injected with MSC. (*p < 0.05, **p < 0.01, ^#^*p* < 0.05 vs Ctrl, ^##^*p* < 0.01 vs Ctrl)

Next, we investigated the mechanism of how hypo-MSC promotes HCC progression. Previous studies have shown that hypoxia could upregulate COX2, which then increased PGE_2_ expression and promoted the growth of esophageal squamous cell carcinoma and colon cancer [18–20]. In HCC, COX2/PGE_2_ pathway also plays a key role in tumor progression [21]. Therefore, we investigated whether COX2 expression was increased in hypo-MSC. Indeed, hypoxia increased the expression of COX2 in MSC, and led to enhanced PGE_2_ secretion (Fig. 2a). To confirm the role of COX2 upregulation in MSC on HCC progression, we knocked down *COX2* in MSC via lentivirus, and found that the PGE_2_ secretion of MSC was significantly reduced under hypoxia condition (Fig. 2b). CCK-8 and Edu incorporation assays showed that the increased cell proliferation of HCC cells was significantly suppressed by COX2 knockdown in MSC (Fig. 2c-e). However, the effect of hypo-MSC on HCC cells growth was not completely eliminated by COX2 knockdown, indicating that other mechanisms might be involved (Fig. 2c-e). Consistently, exogenous PGE_2_ could promote HCC cell growth in a dose dependent manner (Additional file 3: Figure S2). Furthermore, we tested the effects of COX2 knockdown in xenograft models. Both tumor mass and cancer cell proliferation, revealed by Ki-67 staining, were significantly decreased when COX2 was knocked down in MSC (Fig. 2f-g and Additional file 2: Figure S1). Overall, we found that hypoxia led to upregulated COX2 expression in MSC, which promoted HCC progression.

### Hypo-MSC promotes HCC progression through YAP activation

**Fig. 3:**
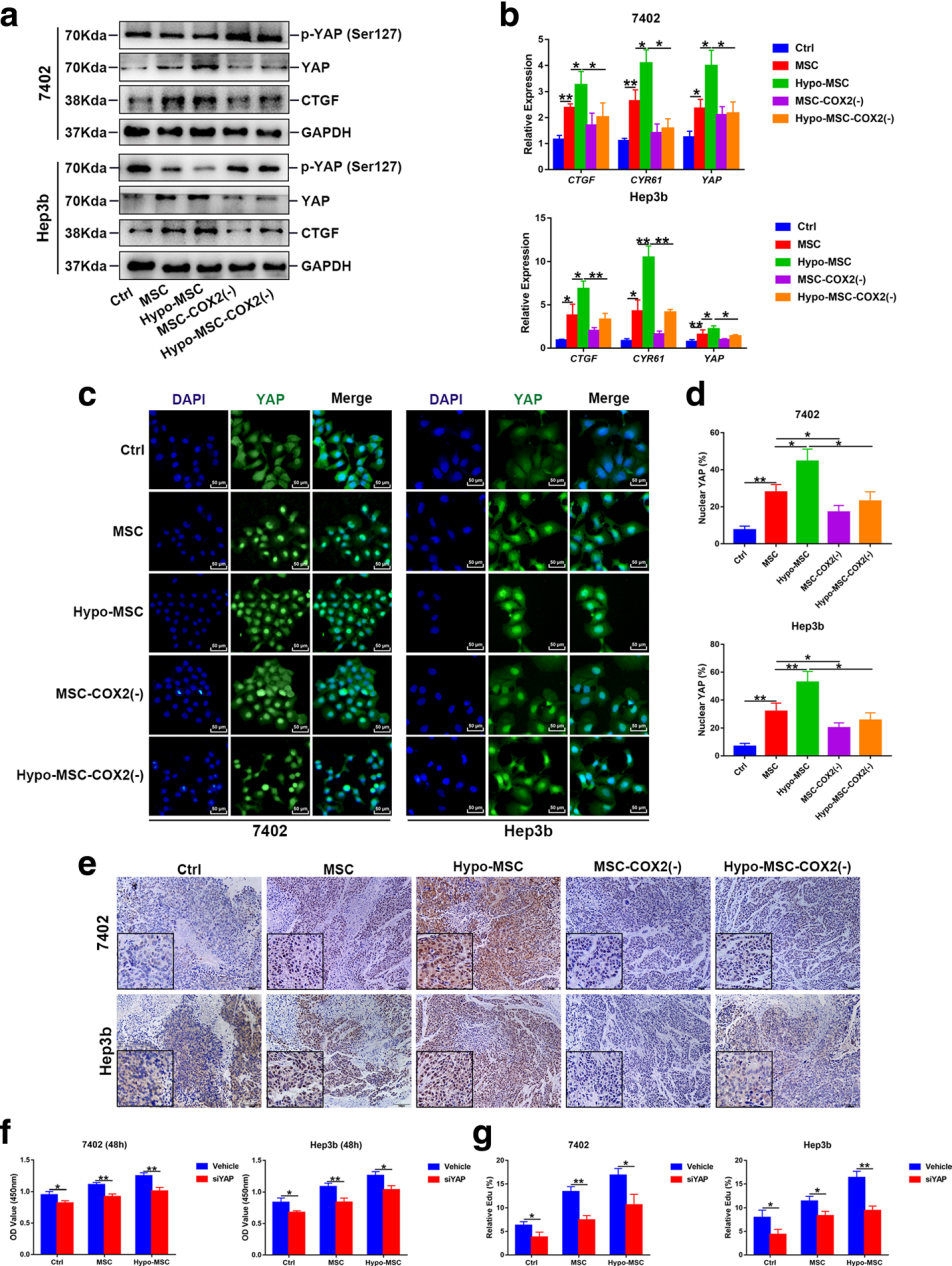
Hypo-MSC activates YAP signaling to promote cell proliferation in HCC cells. **a** Protein levels of YAP and its target CTGF in 7402 and Hep3b under indicated conditions. **b** The mRNA levels of *YAP* and its target genes (*CTGF*, *CYR61*) in 7402 and Hep3b under indicated conditions. **c-d** Immunofluorescence of YAP in in 7402 and Hep3b **c** and quantitative data percentage of cells with nuclear YAP (**d**, *n* = 3). **e** The representative images of YAP in xenograft tumors. **f** The proliferation ability of 7402 and Hep3b after YAP knockdown via siRNA under indicated conditions (*n* = 3). **g** Quantification of Edu positive cells in 7402 and Hep3b cells after YAP knockdown via siRNA under indicated conditions (*n* = 3). (*p < 0.05, **p < 0.01)

YAP plays an important role in the development of HCC [6]. Recent studies have revealed the relationships among COX2, COX2-derived PGE_2_ secretion and YAP activation in cancer development [22–24]. Therefore, we next investigated whether hypo-MSC could also regulate YAP activity in HCC. Western blot showed that YAP was upregulated in HCC cells when the cells were treated with MSC or hypo-MSC-CM (Fig. 3a). Consistently, the mRNA levels of *YAP* and *YAP* target genes, *CTGF* and *CYR61*, were also upregulated in HCC cells by hypo-MSC treatment (Fig. 3b). Immunofluorescence staining confirmed that the translocation of YAP into nucleus in hypo-MSC treated HCC cells (Fig. 3c, d), which was correlated with YAP phosphorylation level (Fig. 3a), as YAP phosphorylation at Ser127 induces its cytoplasmic retention and subsequent degradation [9]. Conversely, knockdown of COX2 in MSC reversed YAP expression and nuclear translocation in HCC cells, even under hypoxia condition (Fig. 3a-d). Consistently, exogenous PGE_2_ showed the similar effect on YAP activation Additional file 4: Figure S3. Moreover, the xenograft tumors showed YAP activation in groups with hypo-MSC co-injection, as indicated by IHC staining (Fig. 3e). Since hypo-MSC could activate YAP in HCC cell lines, we next asked whether YAP was required for hypo-MSC induced cell proliferation. We knocked down YAP in HCC cell lines with siRNA, and found that the increased HCC cell proliferation mediated by hypo-MSC was abolished by YAP knockdown (Fig. 3f). Edu incorporation assays further confirmed this result (Fig. 3g). Collectively, our results suggested that hypo-MSC could activate YAP in HCC cells, leading to increased cell proliferation.

### Hypo-MSC promotes HCC progression through YAP mediated lipogenesis

**Fig. 4:**
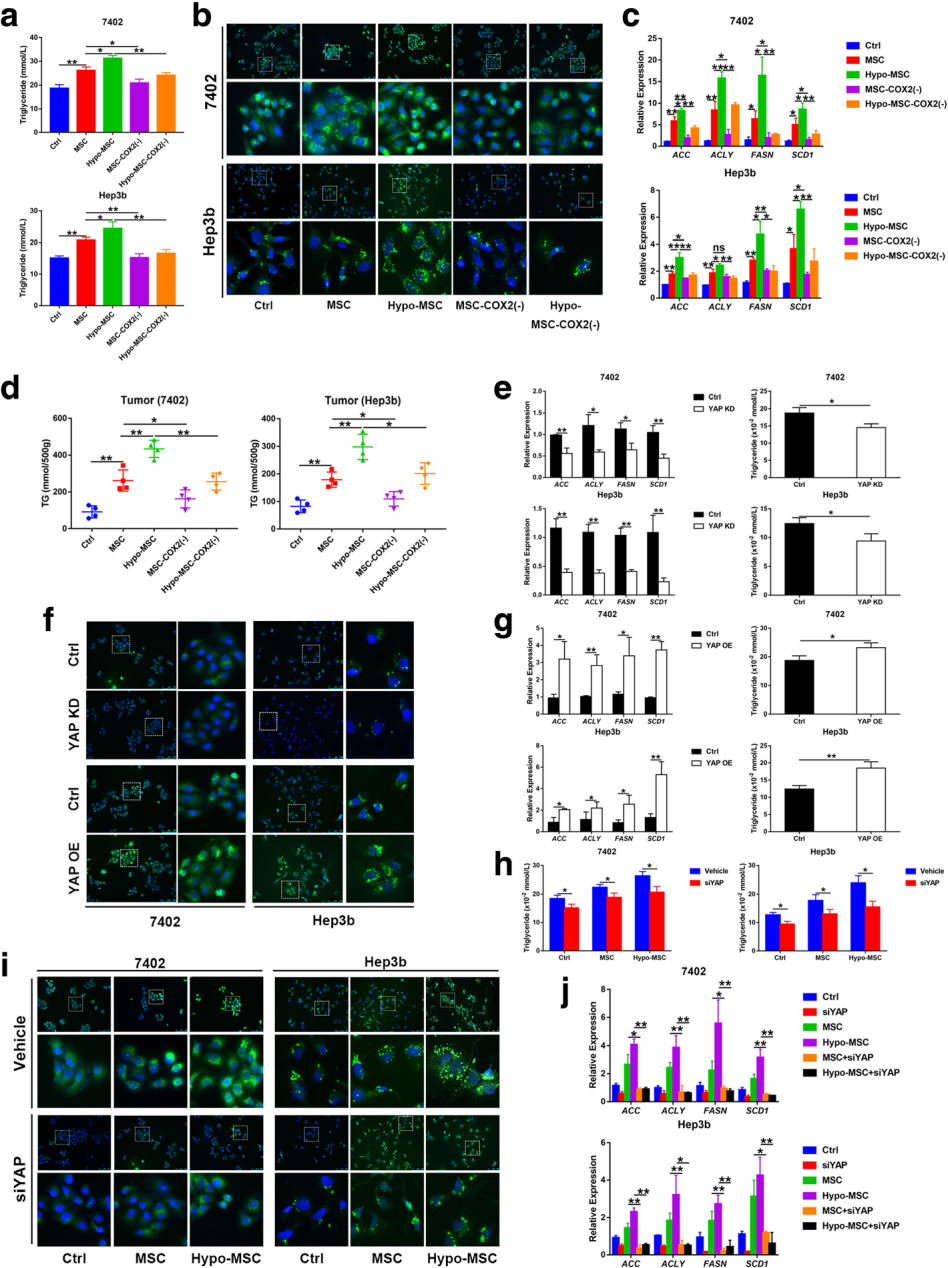
Hypo-MSC enhances lipogenesis via YAP in HCC cell lines. **a** Cellular TG levels were detected in 7402 and Hep3b under indicated conditions (*n* = 3). **b** The content of neutral lipids was detected by with BODIPY 493/503 dye staining in 7402 and Hep3b under indicated conditions. **c** The mRNA levels of lipogenic enzymes in 7402 and Hep3b under indicated conditions (*n* = 3). **d** Levels of TG in xenograft tumors on different groups (*n* = 4). **e-g** Cellular content of TG, neutral lipids and lipogenic enzymes were detected in YAP knockdown (YAP KD) or overexpression (YAP OE) cells. **h** Cellular content of TG were detected in YAP knockdown (siYAP) cells under indicated conditions (*n* = 3). **i** The content of neutral lipids was detected by BODIPY 493/503 dye staining in YAP knockdown (siYAP) cells under indicated conditions. **j** The mRNA levels of lipogenic enzymes in YAP knockdown (siYAP) cells under indicated conditions (*n* = 3). (*p < 0.05, **p < 0.01)

Altered lipid metabolism affects numerous cellular processes, including proliferation, motility, and tumorigenesis [10]. Therefore, to examine whether hypo-MSC can affect lipid metabolism in HCC cells, we evaluated the lipid contents in HCC cell lines when treated with hypo-MSC. Indeed, the intracellular levels of TG were significantly increased in HCC cells when treated with hypo-MSC (Fig. 4a), which was further supported by BODIPY 493/503 staining, suggesting that MSC or hypo-MSC could increase the levels of intracellular neutral lipids in HCC cells (Fig. 4b). Moreover, to test the role of MSC in regulating de novo fatty acid synthesis, we analyzed the expression levels of key lipogenic enzymes in HCC cells, including ATP citrate lyase (ACLY), Acetyl-CoA carboxylase (ACC1), FASN, and Stearoyl-CoA desaturase 1 (SCD1). The results showed that all these lipogenic enzymes were significantly increased in hypo-MSC treated HCC cells (Fig. 4c). Consistently, the TG levels were also increased in the xenograft tumors co-injected with hypo-MSC co-injection (Fig. 4d). Furthermore, the enhanced lipogenesis in HCC cell lines and tumor tissues could be rescued by COX2 knockdown in MSC, even under hypoxia condition.

Previous studies have shown that YAP participates in metabolism regulation, such as promoting glycolysis and lipogenesis [10, 11]. Thus, we further explored the role of YAP on hypo-MSC induced lipogenesis. Knockdown (KD) or overexpression (OE) of YAP in HCC cells significantly decreased or increased lipogenesis (Fig. 4e-g). Also, the hypo-MSC induced lipogenesis was diminished when YAP was knocked down, as shown by intracellular TG levels, BODIPY staining intensity and lipogenic enzymes expression in HCC cells (Fig. 4h-j). Taken together, our results suggested that hypo-MSC promoted HCC progression through YAP mediated lipogenesis.

### YAP enhances lipogenesis via AKT-mTOR-SREBP1 pathway

**Fig. 5:**
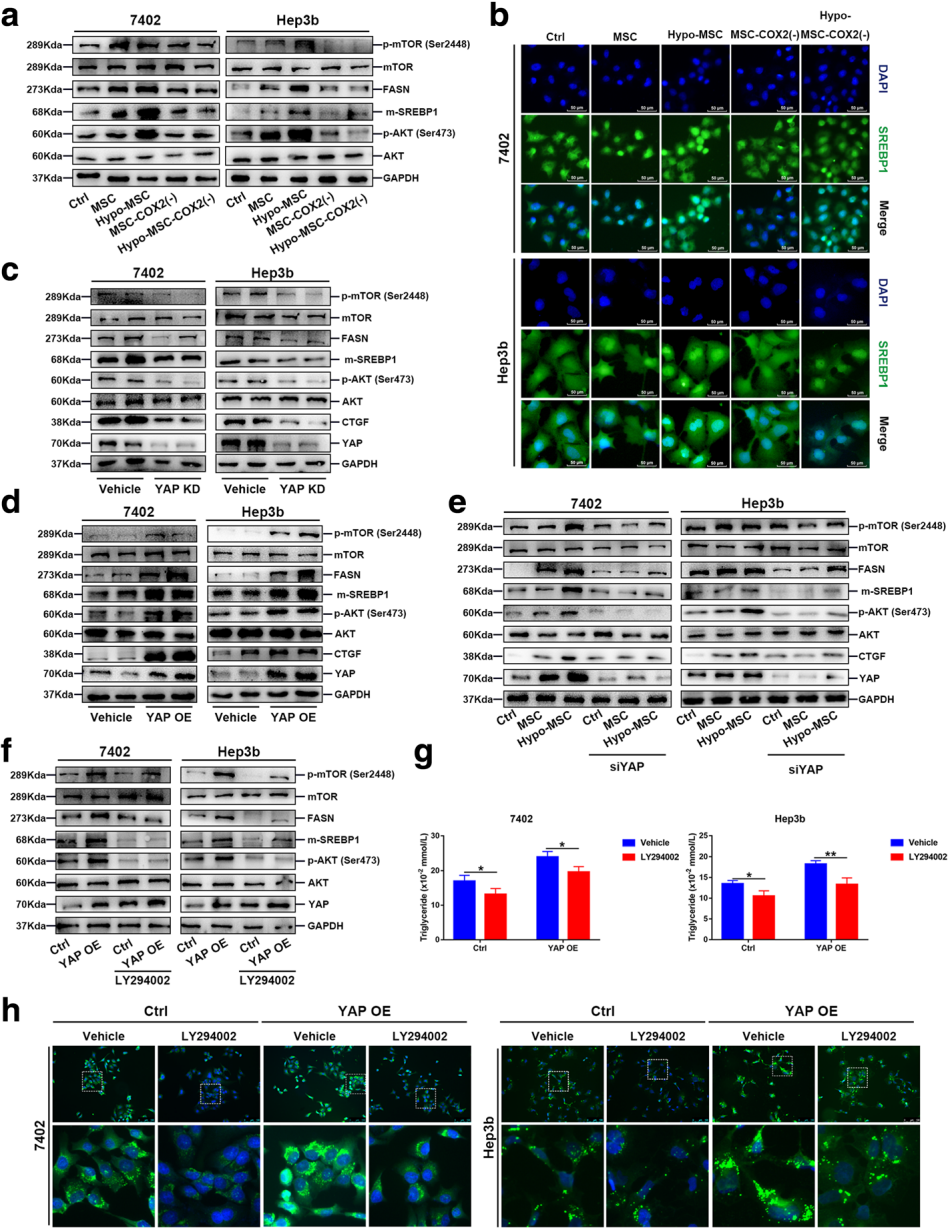
Hypo-MSC enhances lipogenesis via AKT/mTOR/SREBP1 pathway. **a** Protein levels of AKT, mTOR and SREBP1 in indicated cells. **b** Immunofluorescence of SREBP1 in 7402 and Hep3b. **c-d** Protein levels of AKT, mTOR and SREBP1 in YAP KD or YAP OE cells. **e** Protein levels of AKT, mTOR and SREBP1 in YAP knockdown (siYAP) cells under indicated conditions. **f** Protein levels of AKT, mTOR and SREBP1 in YAP OE cells treated with AKT inhibitor LY294002. **g** Cellular content of TG were detected in YAP OE cells treated with AKT inhibitor LY294002 (*n* = 3). **h** The content of neutral lipids was detected by BODIPY 493/503 dye staining in YAP OE cells treated with AKT inhibitor LY294002. (*p < 0.05, **p < 0.01)

Sterol regulatory elementary binding proteins (SREBPs) are important enzymes in regulating fatty acid synthesis [25]. A recent study indicated that SREBP1 contributed to HCC progression by promoting cancer cell growth and metastasis [26]. It has also been shown that LATS2, a core member of Hippo pathway, inhibits SREBP1 and suppresses hepatic cholesterol accumulation [27]. Therefore, we then asked whether hypo-MSC increases SREBP1 expression via YAP to promote lipogenesis. Western blot analysis showed that SREBP1 was upregulated in hypo-MSC treated HCC cells, and COX2 knockdown inhibited SREBP1 expression (Fig. 5a). Moreover, immunofluorescence staining showed that the nuclear accumulation of SREBP1 was increased in hypo-MSC treated HCC cells (Fig. 5b), indicating the active transcription of SRE-containing genes by SREBP1. Consistently, YAP KD or OE in HCC cells significantly inhibited or increased SREBP1 expression (Fig. 5c, d). To test the role of SREBP1 in HCC growth, we knocked down SREBP1 in HCC cells via siRNA, and found that both YAP and SREBP1 were necessary for HCC cells growth, as demonstrated by the CCK-8 and Edu assays (Additional file 5: Figure S4). Furthermore, to confirm the role of SREBP1 in YAP mediated lipogenesis, we treated YAP OE cells with SREBP1 inhibitor, Fatostatin. Fatostatin treatment significantly decreased the intracellular TG levels, staining intensity, and expression of lipogenic enzymes (Additional file 6: Figure S5). These results indicated that the hypo-MSC could induce lipogenesis in HCC cells via YAP mediated SREBP1 upregulation.

Next, we explored the mechanism of how YAP activated SREBP1. As YAP is a transcriptional co-activator, we performed CO-IP to see whether YAP directly binds to SREBP1. However, we did not see directed interaction between YAP and SREBP1 (Additional file 7: Figure S6). Previous studies have demonstrated that the AKT/mTOR pathway plays an important role in regulating fatty acid synthesis [12], and mTOR can promote de novo lipid synthesis through SREBP [28]. Since increased YAP expression in human liver tumors is associated with high levels of p-AKT [29], we hypothesized that in hypo-MSC treated HCC cells, YAP activation activates AKT/mTOR pathway, which then promotes the SREBP1-mediated upregulation of lipogenic enzymes. Western blot analysis showed that the phosphorylation of both AKT and mTOR were significantly increased in cells treated with hypo-MSC, and knockdown of COX2 in MSC could reduce these phosphorylations (Fig. 5a). YAP KD or OE experiments confirmed the regulatory role of YAP in AKT/mTOR pathway (Fig. 5c, d). Also, when YAP was knocked down, hypo-MSC failed to activate AKT/mTOR pathway or SREBP1 expression in HCC cells (Fig. 5e). Furthermore, when we treated cells with PI3K inhibitor, LY294002, the levels of p-AKT and p-mTOR were dramatically decreased in HCC, even under YAP OE conditions (Fig. 5f), and the expression of SREBP1 was remarkably decreased as expected (Fig. 5f). In addition, we also observed reduced levels of intracellular TG and weakened BODIPY staining in cells with LY294002 treatment (Fig. 5g, h). Overall, these results suggested that YAP induced lipogenesis in HCC cells via AKT/mTOR/SREBP1 pathway.

### Hypo-MSC-derived PGE_2_ promotes HCC via prostaglandin E receptor 4 (EP4)

**Fig. 6:**
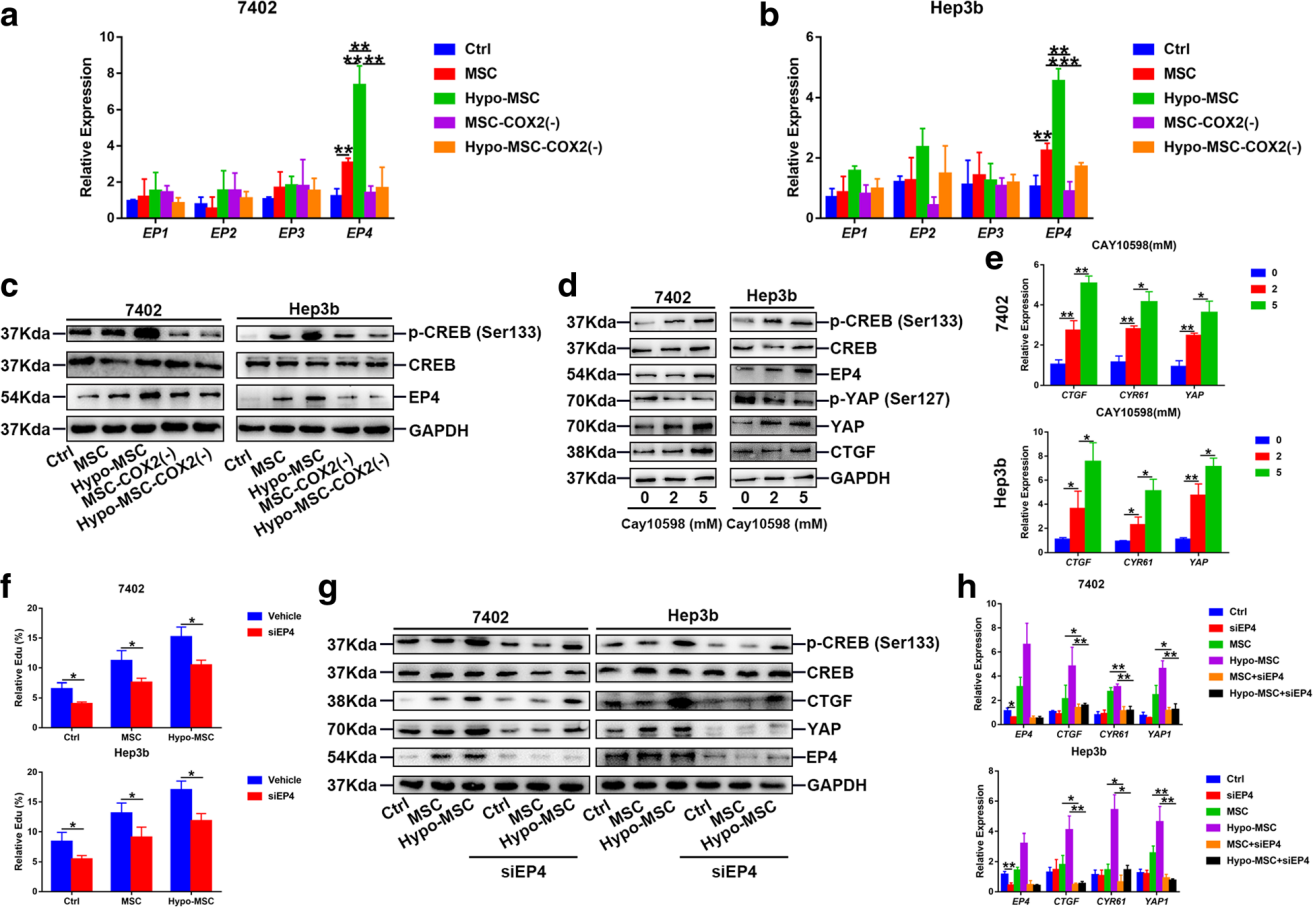
Hypo-MSC activates YAP via EP4. **a-b** The mRNA levels of *EP1*-*EP4* in 7402 and Hep3b under indicated conditions. **c** Protein levels of EP4 and CREB in indicated cells. **d-e** Protein and mRNA levels of YAP and its target genes in cells treated with CAY10598 (EP4 agonist) in indicated dose. **f** Quantification of Edu positive cells in EP4 knockdown cells via siRNA under different condition (*n* = 3). **g-h** Protein and mRNA levels of YAP and its target genes in EP4 knockdown cells via siRNA under indicated conditions. (*p < 0.05, **p < 0.01)

PGE_2_ is known to have four types of receptors: EP1–4. Thus, we next investigated the mechanisms of how hypo-MSC-derived PGE_2_ activated YAP in HCC cells. We found that EP4 was significantly upregulated in hypo-MSC treated HCC cells (Fig. 6a, b), as well as the cAMP response element binding protein (CREB) (Fig. 6c), which has been recently shown to promote YAP activation in HCC [8]. To confirm the role of EP4 in hypo-MSC mediated HCC progression, we used EP4 agonist, Cay10598, to activate EP4 in HCC cells, and found YAP was also activated (Fig. 6d, e). We also knocked down EP4 in HCC cells using siRNA, and found that hypo-MSC was unable to improve HCC cell proliferation (Fig. 6f), and the expression of *YAP* and its target genes were suppressed in HCC cells (Fig. 6g, h). Consistently, exogenous PGE_2_ increased EP4 expression and failed to activate YAP when EP4 was knocked down (Additional file 8: Figure S7).

**Fig. 7:**
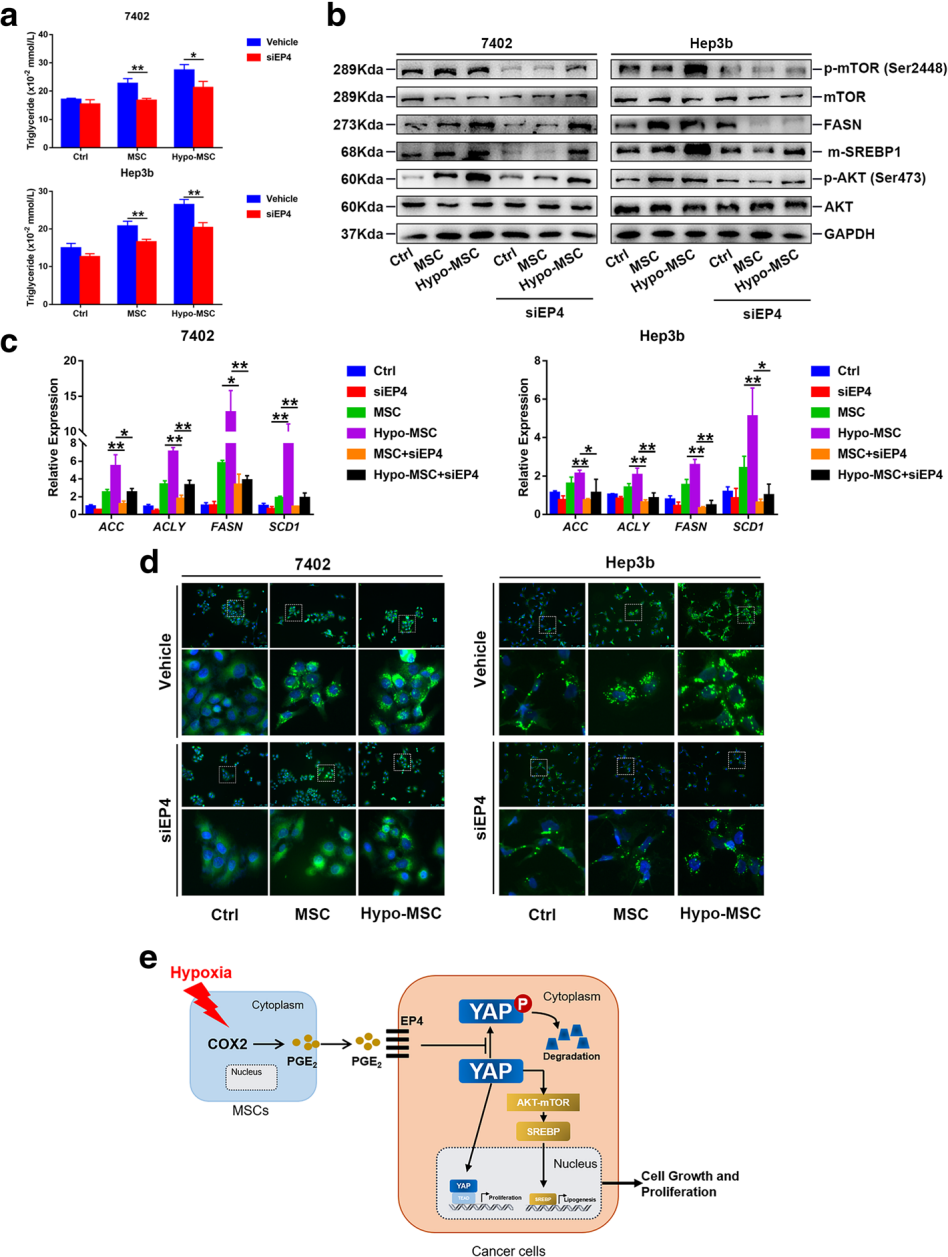
Hypo-MSC promotes lipogenesis via EP4 mediated YAP activation in cells. **a** Cellular levels of TG in EP4 knockdown cells under indicated conditions (*n* = 3). **b** Protein levels of AKT, mTOR and SREBP1 in EP4 knockdown cells under indicated conditions. **c** The mRNA levels of lipogenic enzymes in EP4 knockdown cells under indicated conditions (*n* = 3). **d** The content of neutral lipids in EP4 knockdown cells under indicated conditions. **e** Proposed model for hypo-MSC on HCC progression. (*p < 0.05, **p < 0.01)

Next, we explored the role of EP4 in hypo-MSC induced lipogenesis. Knocking down EP4 significantly reduced the intracellular TG levels, BODIPY staining intensity, and the expression of lipogenic enzymes in HCC cells (Fig. 7a-d). Moreover, the levels of SREBP1, p-AKT and p-mTOR were decreased with EP4 knockdown (Fig. 7b). We also treated the HCC cells with EP4 antagonist GW627368X, and found similar effects on YAP and lipogenesis as EP4 siRNA (Additional file 9: Figure S8). Altogether, our results indicated that hypo-MSC promoted YAP and lipogenesis via PGE_2_/EP4 axis.

## Discussion

As an important part of TME, MSC have been shown to promote cancer progression by numerous studies [1]. Recent studies have also indicated that tumorigenesis heavily relies on the reciprocal interactions between tumor cells and the surrounding stroma [2, 30]. Therefore, identifying the critical pathway involved in this cross-talk could potentially improve the efficacy of cancer treatment [31, 32]. Hypoxia is an important hallmark of solid tumors, and is associated with tumor progression [33]. In this study, we further demonstrated the critical role of hypoxia in MSC mediated HCC progression.

Previous studies have confirmed the formation of paracrine loop between MSC and tumor cells, which resulting in tumor progression and metastasis [34]. Thus, we hypothesized that the cytokines secreted by MSC might be changed under hypoxia condition, leading to increased HCC cell proliferation. COX2 has been shown to induce tumor initiation, progression and angiogenesis in different cancer types. For example, hepatic COX2 overexpression induced spontaneous hepatocellular carcinoma formation in mice [35, 36]. However, most of these studies focused on the role of COX2 in parenchymal cells. Recently, several studies revealed the role of COX2 in noparenchymal cells residing in TME. For example, the myeloid-derived suppressor cells (MDSCs) and macrophages could promote cancer progression through secreting PGE_2_, a key product of COX2 [37, 38]. Moreover, COX2 expression could be upregulated by hypoxia [20, 39], which is consistent with our findings. In this study, we found that hypoxia could increase COX2 expression in MSC, which led to enhanced PGE_2_ secretion and HCC progression. However, knockdown of COX2 did not completely eliminate the effect on HCC progression under hypoxia condition, indicating that other cytokines in MSC might also be upregulated under hypoxia condition and promote HCC growth.

Although many evidences have pointed out that YAP is a key regulator in many types of solid tumors, the role of YAP activation in HCC has not been fully elucidated. In the present study, we observed increased expression and nuclear accumulation of YAP in HCC cells treated with hypo-MSC. Knockdown of COX2 in MSC suppressed the expression of YAP and impaired the effect of MSC on HCC cell proliferation, even under hypoxia condition. This result is consistent with the previous evidence that PGE_2_ can activate YAP in colon cancer [40]. Moreover, depletion of YAP significantly impaired the effect of MSC on HCC cell proliferation, demonstrating the importance of YAP in hypo-MSC mediated HCC cell proliferation.

Currently, it has been suggested that YAP is an integrator of metabolic cues and cell growth signals [13]. In HCC, the high mobility group box-1 protein (HMGB1)-YAP-dependent aerobic glycolysis played an important role in tumor growth [41]. Also, the effects of YAP on inflammation have also been implicated in NASH progression, indicating that YAP might participate in liver lipid metabolism [42, 43]. Consistent with the previous results, our study demonstrated that hypo-MSC promoted lipogenesis in HCC cells via YAP and SREBP1, which then upregulated the key lipogenic enzymes. Several studies have suggested that YAP regulates AKT signaling components, like PI3K, AKT and PTEN [44–47]; on the other hand, MST1, a Hippo pathway component, inhibited AKT activity in *Drosophila* [47]. In human liver tumors, increased expression of YAP is associated with high levels of p-AKT [29], and recent study has showed that Hippo pathway prevented hepatic steatosis and liver tumors by suppressing the insulin receptor substrate (IRS)/AKT signaling [43]. Our study confirmed that YAP activated AKT/mTOR signaling, which then activated SREBP1 and promoted lipogenesis.

PGE_2_ exerts its physiological functions by binding to specific receptors (EP1–4). In this study, we confirmed that hypo-MSC derived PGE_2_ enhanced cell proliferation via EP4, which activated YAP and the YAP mediated lipogenesis. Consistently, knockdown of EP4 or EP4 antagonists inhibited, while EP4 agonists promoted, the cell proliferation and YAP activation.

## Conclusion

Our work demonstrated that hypo-MSC played a pivotal role in HCC progression through the COX2/PGE_2_/EP4/YAP axis. In particular, our data showed that the YAP activation in HCC cells activated AKT/mTOR/SREBP1 pathway, which then enhanced the lipogenesis and accelerated the growth of HCC cells (Fig. 7e). Thus, our findings characterize the role of cross-talk between MSC and HCC cells in HCC development, providing new insights into the mechanisms underlying the interactions between TME and HCC cells. The newly identified mechanism could potentially serve as a target for HCC therapy.

## Additional files

Additional file 1: **Table S1.** Antibodies for immunoblots and immunohistochemistry. **Table S2.** Sequence of primers of qRT-PCR used in experiment. **Table S3.** Primers of siRNAs. **Table S4.** Sequence of lentivirus. (DOCX 17 kb)

Additional file 2: **Figure S1.** CD90 staining of MSC in xenograft tumors. (DOCX 618 kb)

Additional file 3: **Figure S2.** Exogenous PGE_2_ promotes HCC cell proliferation. (a) The proliferation ability of 7402 and Hep3b treated with PGE_2_ in indicated dose. (b-c) Representative images and quantification of Edu positive cells in 7402 and Hep3b cells treated with PGE_2_ in indicated dose (*n* = 3). (*p < 0.05). (DOCX 397 kb)

Additional file 4: **Figure S3.** Exogenous PGE_2_ activates YAP in HCC cell lines. (a) Protein levels of YAP and its target CTGF in 7402 and Hep3b cells treated with PGE_2_ in indicated dose. (b-c) Immunofluorescence of YAP in in 7402 and Hep3b cells and quantitative data percentage of cells with nuclear YAP in cells treated with PGE_2_ in indicated dose (*n* = 3). (d) The mRNA levels of *YAP* and its target genes (*CTGF*, *CYR61*) in 7402 and Hep3b cells treated with PGE_2_ in indicated dose. (*p < 0.05, **p < 0.01). (DOCX 512 kb)

Additional file 5: **Figure S4.** The role of SREBP1 in cell proliferation. (a) Protein levels of SREBP1 in 7402 and Hep3b which transfected with siRNA. (b) The proliferation ability of 7402 and Hep3b after SREBP1 knockdown via siRNA under indicated conditions (*n* = 3). (c) Quantification of Edu positive cells in 7402 and Hep3b cells after SREBP1 knockdown via siRNA under indicated conditions (*n* = 3). (d) The proliferation ability of 7402 and Hep3b after SREBP1 knockdown via siRNA in normal or YAP OE cells (*n* = 3). (e) Quantification of Edu positive cells in 7402 and Hep3b cells after SREBP1 knockdown via siRNA in normal or YAP OE cells (*n* = 3). (*p < 0.05, **p < 0.01). (DOCX 221 kb)

Additional file 6: **Figure S5.** YAP regulates lipogenesis in the presence of SREBP1. (a) Cellular TG levels in YAP OE cells treated with SREBP1 inhibitor, Fatostatin. (b) The mRNA levels of lipogenic enzymes in cells treated with Fatostatin (*n* = 3). (c) The content of neutral lipids in cells treated with Fatostatin. (*p < 0.05, **p < 0.01). (DOCX 494 kb)

Additional file 7: ** Figure S6.** The interaction between SREBP1 and YAP was examined by CO-IP. (DOCX 91 kb)

Additional file 8: ** Figure S7.** PGE_2_ activates YAP via EP4 to promote cell proliferation. (a) the mRNA levels of *EP1*-*EP4* in cells treated with PGE_2_. (b) Expression of EP4 and CREB in cells treated with PGE_2_. (c) Expression of EP4, CREB and YAP in EP4 knockdown cells treated with PGE_2_. (d) Quantification of Edu positive cells in EP4 knockdown cells treated with PGE_2_. (e) The mRNA levels of *YAP* and its target genes in EP4 knockdown cells treated with PGE_2_. (*p < 0.05, **p < 0.01, ***p < 0.001). (DOCX 432 kb)

Additional file 9: **Figure S8.** The role of GW627368X (EP4 inhibitor, EP4i) on hypo-MSC mediated cell proliferation and lipogenesis. (a) Quantification of Edu positive cells in cells treatment of EP4i under indicated conditions. (b) Expression of YAP in cells treatment of EP4i under indicated conditions. (c) The mRNA levels of *YAP* and its target genes in cells treatment of EP4i under indicated conditions. (d) Expression of AKT, mTOR and SREBP1 in cells treatment of EP4i under indicated conditions. (e) Cellular TG levels in cells treatment of EP4i under indicated conditions. (f) The content of neutral lipids in cells treatment of EP4i under indicated conditions. EP4i: EP4 inhibitor. (*p < 0.05, **p < 0.01). (DOCX 1210 kb)

## Abbreviations

ACC1: Acetyl-CoA carboxylase
ACLY: ATP citrate lyase
CCK-8: Cell counting kit-8
CCL5: CC-chemokine ligand 5
COX2: Cyclooxygenase 2
CREB: CAMP response element binding protein
CXCL10: CXC-chemokine ligand 10
EP4: Prostaglandin E receptor 4
FASN: Fatty acid synthase
HCC: Hepatocellular carcinoma
MSC: Mesenchymal stem cell
PGE_2_: Prostaglandin E_2_
qRT-PCR: quantitative real-time PCR
SCD1: Stearoyl-CoA desaturase 1
siRNA: small interfering RNA
SREBP: Sterol regulatory elementary binding proteins
TG: Triacylglycerol
TGFβ: Transforming growth factor-β
TME: Tumor microenvironment
YAP: Yes-associated protein
