# Supplementary material for: The hypoxia conditioned mesenchymal stem cells promote hepatocellular carcinoma progression through YAP mediated lipogenesis reprogramming
Source: J Exp Clin Cancer Res. 2019 May 29;38:228. doi: 10.1186/s13046-019-1219-7 (PMC6540399; doi:10.1186/s13046-019-1219-7)
Supplement: Supplementary file 1 — Table S1. Antibodies for immunoblots and immunohistochemistry. Table S2. Sequence of primers of qRT-PCR used in experiment. Table S3. Primers of siRNAs. Table S4. Sequence of lentivirus. (DOCX 17 kb) [file 13046_2019_1219_MOESM1_ESM.docx]

**Table S1. Antibodies for immunoblots and immunohistochemistry**

| Antibodies | Company | Dilution | Catalogue number |
| --- | --- | --- | --- |
| GAPDH | Abcam | 1:10000 | ab181603 |
| p-YAP (Ser127) | Abcam | 1:1000 | ab76252 |
| YAP | Abcam | 1:1000 (WB)  1:100 (IF,IHC) | ab205270 |
| CTGF | Santa Cruz Technology | 1:200 | sc-373936 |
| p-MTOR (Ser2448) | Cell Signaling Technology | 1:1000 | 5536 |
| MTOR | Cell Signaling Technology | 1:1000 | 2983 |
| p-AKT (Ser473) | Abcam | 1:5000 | ab81283 |
| AKT | Abcam | 1:5000 | ab179463 |
| FASN | Abcam | 1:3000 | ab128870 |
| SREBP1c | Santa Cruz Technology | 1:200 (WB)  1:50 (IF) | sc-13551 |
| p-CREB (Ser133) | Abcam | 1:1000 | ab32096 |
| CREB | Cell Signaling Technology | 1:1000 | 9197 |
| COX2 | Abcam | 1:1000 | ab179800 |
| EP4 | Santa Cruz Technology | 1:200 | sc-55596 |
| Ki67 | Abcam | 1:250 (IHC) | ab16667 |
| CD90 | Abcam | 1:200 (IHC) | ab133350 |

**Table S2. Sequence of primers used in experiments**

| Gene | Forward | Reverse |
| --- | --- | --- |
| 18S | AACCCGTTGAACCCCATT | CCATCCAATCGGTAGTAGCG |
| CTGF | CAGCATGGACGTTCGTCTG | AACCACGGTTTGGTCCTTGG |
| CYR61 | CTCGCCTTAGTCGTCACCC | CGCCGAAGTTGCATTCCAG |
| YAP | TAGCCCTGCGTAGCCAGTTA | TCATGCTTAGTCCACTGTCTGT |
| ACC | CTCTCACGCTCAAGTCACCA | ACTTGGTTATGGCGAAGCTC |
| ACLY | CAGCAGGACAGCATCTTTTTC | TGGACTTGGGACTGAATCTTG |
| FASN | ACTCCATGTTTGGTGTTTGTC | TGGAGATCACATGCGGTTTA |
| SCD1 | CCCCACCTACAAGGATAAGGA | CACGAGCCCATTCATAGACAT |
| EP1 | AGCTTGTCGGTATCATGGTGG | AAGAGGCGAAGCAGTTGGC |
| EP2 | CGATGCTCATGCTCTTCGC | GGGAGACTGCATAGATGACAGG |
| EP3 | CGCCTCAACCACTCCTACAC | GACACCGATCCGCAATCCTC |
| EP4 | CCGGCGGTGATGTTCATCTT | CCCACATACCAGCGTGTAGAA |

**Table S3. Primers of siRNAs**

| siEP4 | CTGAGGACTTTGCGAATAT |
| --- | --- |
| siYAP | GAGATGGAATGAACATAGA |
| siSREBP1 | CGGAGAAGCTGCCTATCAA |

**Table S4. Sequence of lentiviral RNA**

| COX2 | CCGGGCTGAATTTAACACCCTCTATCTCGAGATAGAGGGTGTTAAATTCAGCTTTTT |
| --- | --- |
| YAP | ATGGATCCCGGGCAGCAGCCGCCGCCTCAACCGGCCCCCCAGGGCCAAGGGCAGCCGCCTTCGCAGCCCCCGCAGGGGCAGGGCCCGCCGTCCGGACCCGGGCAACCGGCACCCGCGGCGACCCAGGCGGCGCCGCAGGCACCCCCCGCCGGGCATCAGATCGTGCACGTCCGCGGGGACTCGGAGACCGACCTGGAGGCGCTCTTCAACGCCGTCATGAACCCCAAGACGGCCAACGTGCCCCAGACCGTGCCCATGAGGCTCCGGAAGCTGCCCGACTCCTTCTTCAAGCCGCCGGAGCCCAAATCCCACTCCCGACAGGCCAGTACTGATGCAGGCACTGCAGGAGCCCTGACTCCACAGCATGTTCGAGCTCATTCCTCTCCAGCTTCTCTGCAGTTGGGAGCTGTTTCTCCTGGGACACTGACCCCCACTGGAGTAGTCTCTGGCCCAGCAGCTACACCCACAGCTCAGCATCTTCGACAGTCTTCTTTTGAGATACCTGATGATGTACCTCTGCCAGCAGGTTGGGAGATGGCAAAGACATCTTCTGGTCAGAGATACTTCTTAAATCACATCGATCAGACAACAACATGGCAGGACCCCAGGAAGGCCATGCTGTCCCAGATGAACGTCACAGCCCCCACCAGTCCACCAGTGCAGCAGAATATGATGAACTCGGCTTCAGGTCCTCTTCCTGATGGATGGGAACAAGCCATGACTCAGGATGGAGAAATTTACTATATAAACCATAAGAACAAGACCACCTCTTGGCTAGACCCAAGGCTTGACCCTCGTTTTGCCATGAACCAGAGAATCAGTCAGAGTGCTCCAGTGAAACAGCCACCACCCCTGGCTCCCCAGAGCCCACAGGGAGGCGTCATGGGTGGCAGCAACTCCAACCAGCAGCAACAGATGCGACTGCAGCAACTGCAGATGGAGAAGGAGAGGCTGCGGCTGAAACAGCAAGAACTGCTTCGGCAGGCAATGCGGAATATCAATCCCAGCACAGCAAATTCTCCAAAATGTCAGGAGTTAGCCCTGCGTAGCCAGTTACCAACACTGGAGCAGGATGGTGGGACTCAAAATCCAGTGTCTTCTCCCGGGATGTCTCAGGAATTGAGAACAATGACGACCAATAGCTCAGATCCTTTCCTTAACAGTGGCACCTATCACTCTCGAGATGAGAGTACAGACAGTGGACTAAGCATGAGCAGCTACAGTGTCCCTCGAACCCCAGATGACTTCCTGAACAGTGTGGATGAGATGGATACAGGTGATACTATCAACCAAAGCACCCTGCCCTCACAGCAGAACCGTTTCCCAGACTACCTTGAAGCCATTCCTGGGACAAATGTGGACCTTGGAACACTGGAAGGAGATGGAATGAACATAGAAGGAGAGGAGCTGATGCCAAGTCTGCAGGAAGCTTTGAGTTCTGACATCCTTAATGACATGGAGTCTGTTTTGGCTGCCACCAAGCTAGATAAAGAAAGCTTTCTTACATGGTTA |
