# Supplementary figures and images for: The hypoxia conditioned mesenchymal stem cells promote hepatocellular carcinoma progression through YAP mediated lipogenesis reprogramming
Source: J Exp Clin Cancer Res. 2019 May 29;38:228. doi: 10.1186/s13046-019-1219-7 (PMC6540399; doi:10.1186/s13046-019-1219-7)

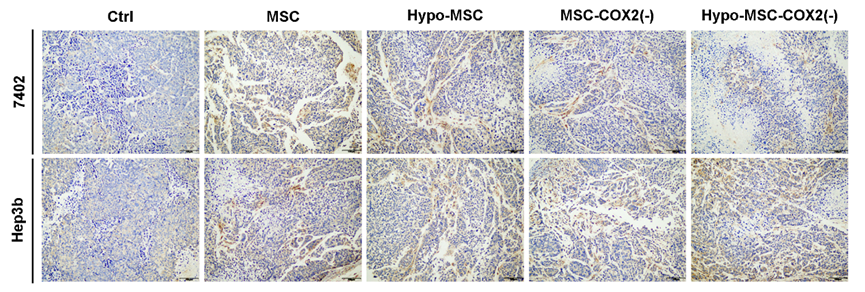


**Figure S1.** CD90 staining of MSC in xenograft tumors.

Supplement: Supplementary file 2 — Figure S1. CD90 staining of MSC in xenograft tumors. (DOCX 618 kb) [file 13046_2019_1219_MOESM2_ESM.docx]

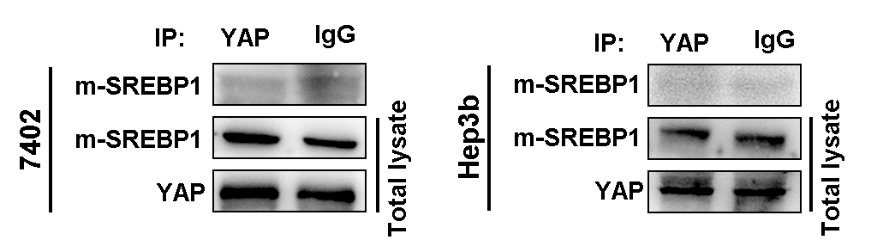


**Figure S6.** The interaction between SREBP1 and YAP was examined by CO-IP.

Supplement: Supplementary file 7 — Figure S6. The interaction between SREBP1 and YAP was examined by CO-IP. (DOCX 91 kb) [file 13046_2019_1219_MOESM7_ESM.docx]
