# Supplementary material for: The hypoxia conditioned mesenchymal stem cells promote hepatocellular carcinoma progression through YAP mediated lipogenesis reprogramming
Source: J Exp Clin Cancer Res. 2019 May 29;38:228. doi: 10.1186/s13046-019-1219-7 (PMC6540399; doi:10.1186/s13046-019-1219-7)
Supplement: Supplementary file 3 — Figure S2. Exogenous PGE2 promotes HCC cell proliferation. (a) The proliferation ability of 7402 and Hep3b treated with PGE2 in indicated dose. (b-c) Representative images and quantification of Edu positive cells in 7402 and Hep3b cells treated with PGE2 in indicated dose (n = 3). (*p < 0.05). (DOCX 397 kb) [file 13046_2019_1219_MOESM3_ESM.docx]

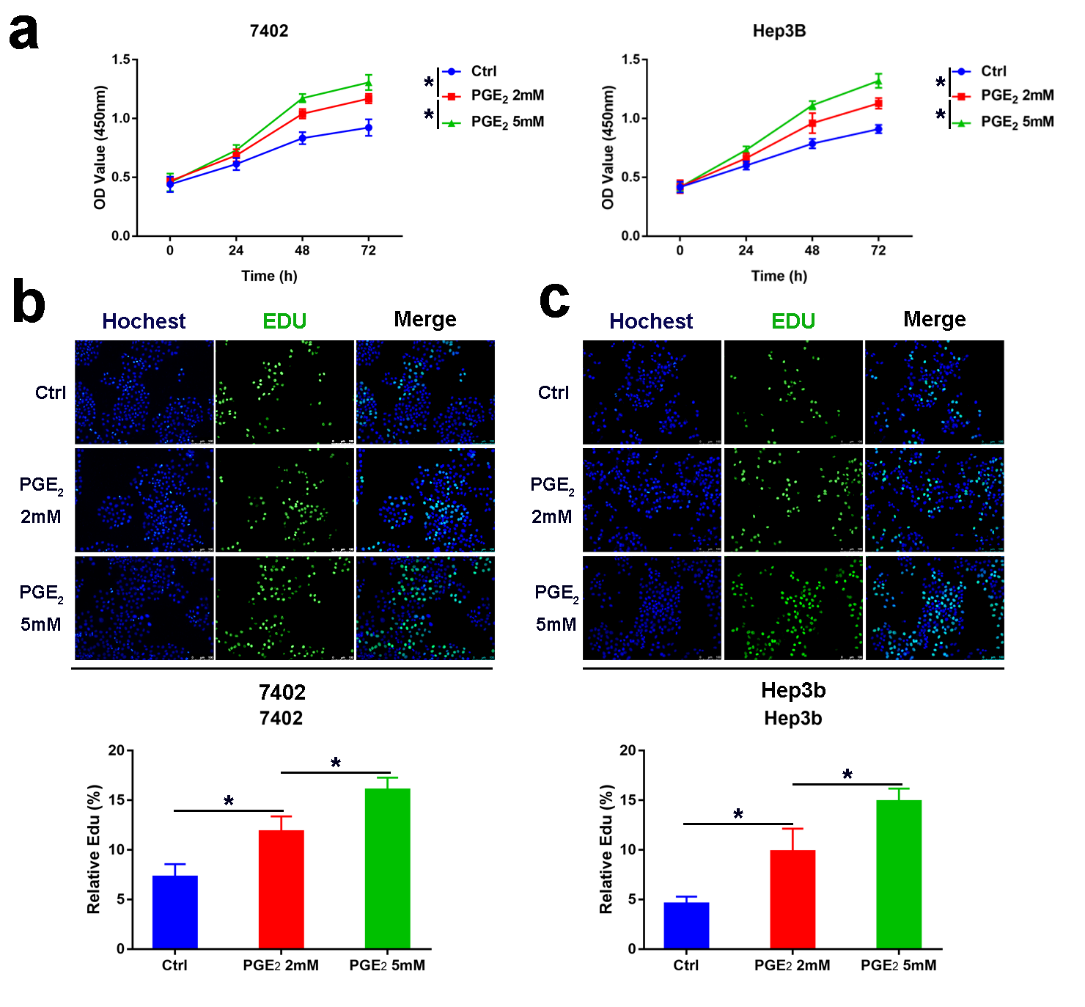


**Figure S2.** Exogenous PGE_2_ promotes HCC cells proliferation. (a) The proliferation ability of 7402 and Hep3b treated with PGE_2_ in indicated dose. (b-c) Representative images and quantification of Edu positive cells in 7402 and Hep3b cells treated with PGE_2_ in indicated dose (n=3). (*p<0.05).
