# Supplementary material for: The hypoxia conditioned mesenchymal stem cells promote hepatocellular carcinoma progression through YAP mediated lipogenesis reprogramming
Source: J Exp Clin Cancer Res. 2019 May 29;38:228. doi: 10.1186/s13046-019-1219-7 (PMC6540399; doi:10.1186/s13046-019-1219-7)
Supplement: Supplementary file 4 — Figure S3. Exogenous PGE2 activates YAP in HCC cell lines. (a) Protein levels of YAP and its target CTGF in 7402 and Hep3b cells treated with PGE2 in indicated dose. (b-c) Immunofluorescence of YAP in in 7402 and Hep3b cells and quantitative data percentage of cells with nuclear YAP in cells treated with PGE2 in indicated dose (n = 3). (d) The mRNA levels of YAP and its target genes (CTGF, CYR61) in 7402 and Hep3b cells treated with PGE2 in indicated dose. (*p < 0.05, **p < 0.01). (DOCX 512 kb) [file 13046_2019_1219_MOESM4_ESM.docx]

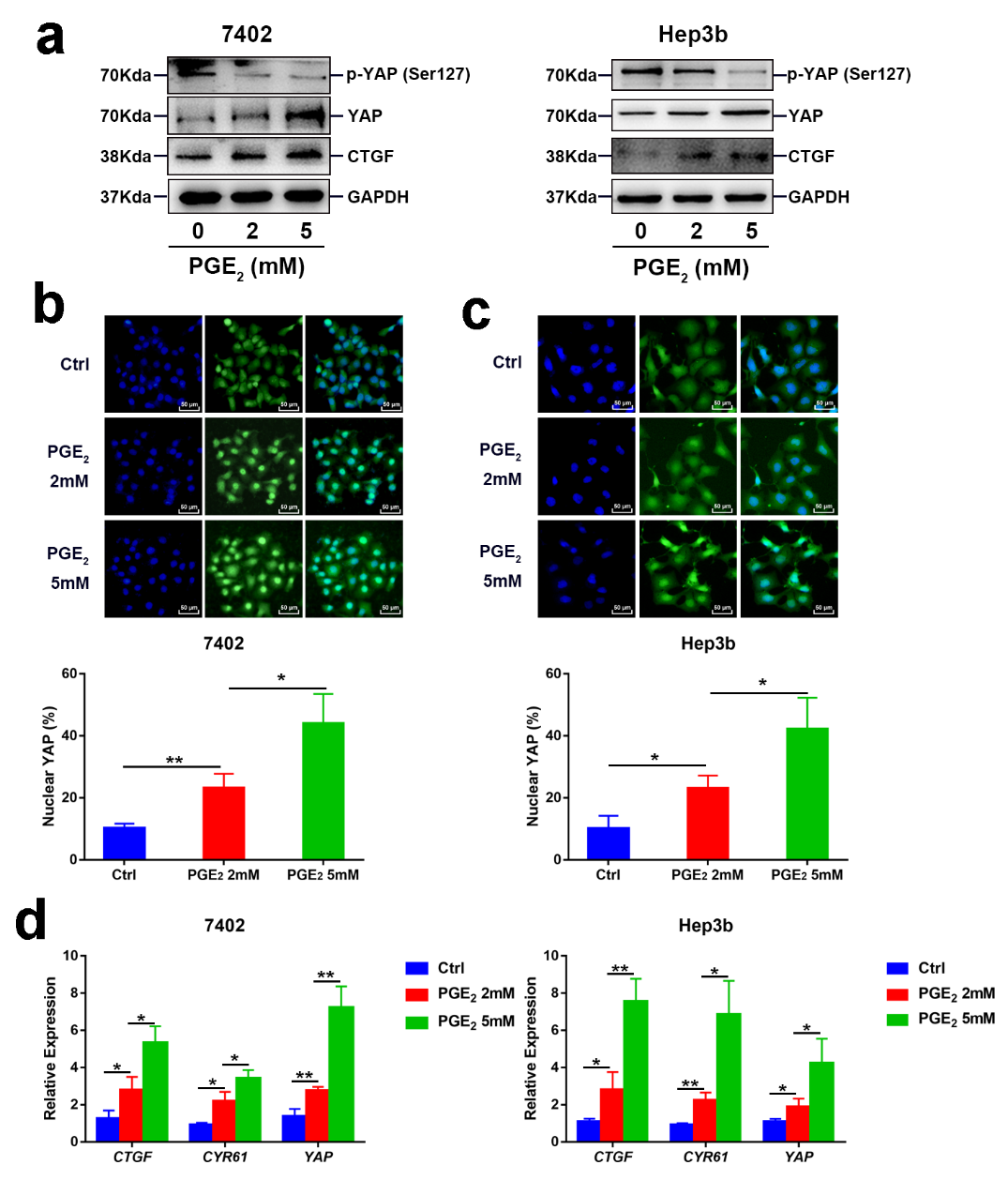


**Figure S3.** Exogenous PGE_2_ activates YAP in HCC cell lines. (a) Protein levels of YAP and its target CTGF in 7402 and Hep3b cells treated with PGE_2_ in indicated dose. (b-c) Immunofluorescence of YAP in in 7402 and Hep3b cells and quantitative data percentage of cells with nuclear YAP in cells treated with PGE_2_ in indicated dose (n=3). (d) The mRNA levels of *YAP* and its target genes (*CTGF*, *CYR61*) in 7402 and Hep3b cells treated with PGE_2_ in indicated dose. (*p<0.05, **p<0.01).
