# Supplementary material for: The hypoxia conditioned mesenchymal stem cells promote hepatocellular carcinoma progression through YAP mediated lipogenesis reprogramming
Source: J Exp Clin Cancer Res. 2019 May 29;38:228. doi: 10.1186/s13046-019-1219-7 (PMC6540399; doi:10.1186/s13046-019-1219-7)
Supplement: Supplementary file 5 — Figure S4. The role of SREBP1 in cell proliferation. (a) Protein levels of SREBP1 in 7402 and Hep3b which transfected with siRNA. (b) The proliferation ability of 7402 and Hep3b after SREBP1 knockdown via siRNA under indicated conditions (n = 3). (c) Quantification of Edu positive cells in 7402 and Hep3b cells after SREBP1 knockdown via siRNA under indicated conditions (n = 3). (d) The proliferation ability of 7402 and Hep3b after SREBP1 knockdown via siRNA in normal or YAP OE cells (n = 3). (e) Quantification of Edu positive cells in 7402 and Hep3b cells after SREBP1 knockdown via siRNA in normal or YAP OE cells (n = 3). (*p < 0.05, **p < 0.01). (DOCX 221 kb) [file 13046_2019_1219_MOESM5_ESM.docx]

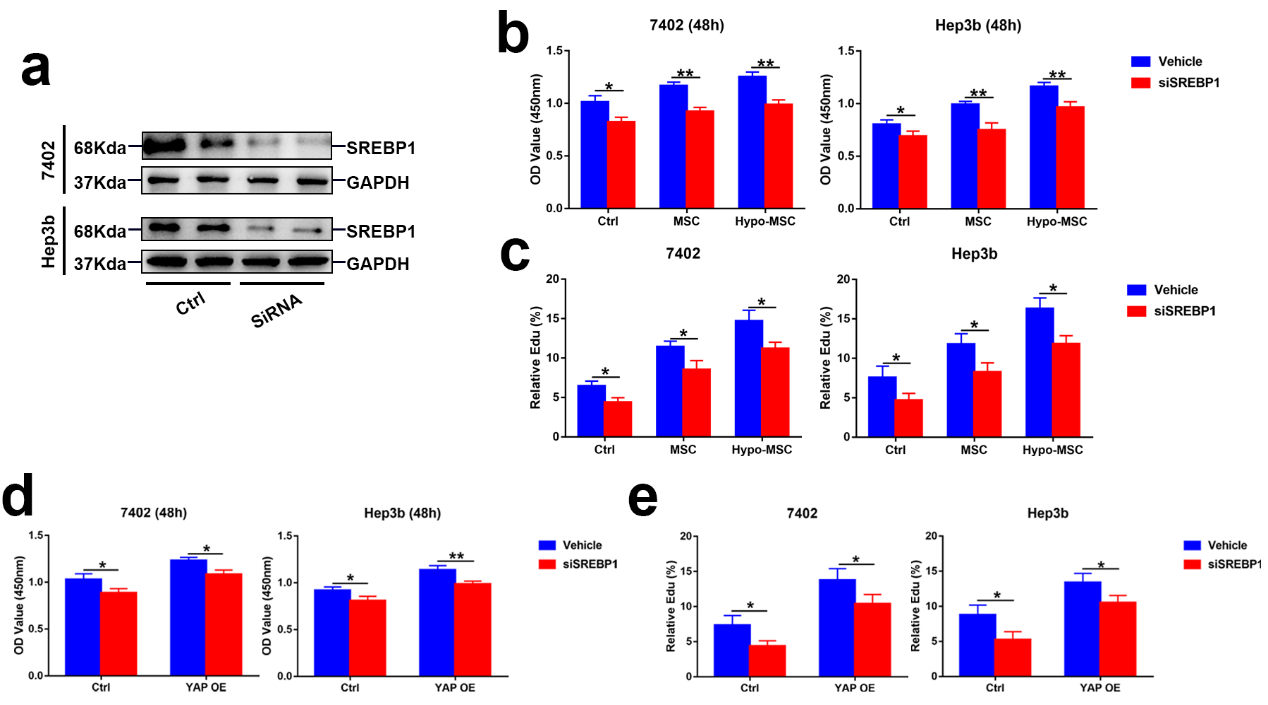
 **Figure S4.** The role of SREBP1 in cell proliferation. (a) Protein levels of SREBP1 in 7402 and Hep3b which transfected with siRNA. (b) The proliferation ability of 7402 and Hep3b after SREBP1 knockdown via siRNA under indicated conditions (n=3). (c) Quantification of Edu positive cells in 7402 and Hep3b cells after SREBP1 knockdown via siRNA under indicated conditions (n=3). (d) The proliferation ability of 7402 and Hep3b after SREBP1 knockdown via siRNA in normal or YAP OE cells (n=3). (e) Quantification of Edu positive cells in 7402 and Hep3b cells after SREBP1 knockdown via siRNA in normal or YAP OE cells (n=3). (*p<0.05, **p<0.01).
