# Supplementary material for: The hypoxia conditioned mesenchymal stem cells promote hepatocellular carcinoma progression through YAP mediated lipogenesis reprogramming
Source: J Exp Clin Cancer Res. 2019 May 29;38:228. doi: 10.1186/s13046-019-1219-7 (PMC6540399; doi:10.1186/s13046-019-1219-7)
Supplement: Supplementary file 6 — Figure S5. YAP regulates lipogenesis in the presence of SREBP1. (a) Cellular TG levels in YAP OE cells treated with SREBP1 inhibitor, Fatostatin. (b) The mRNA levels of lipogenic enzymes in cells treated with Fatostatin (n = 3). (c) The content of neutral lipids in cells treated with Fatostatin. (*p < 0.05, **p < 0.01). (DOCX 494 kb) [file 13046_2019_1219_MOESM6_ESM.docx]

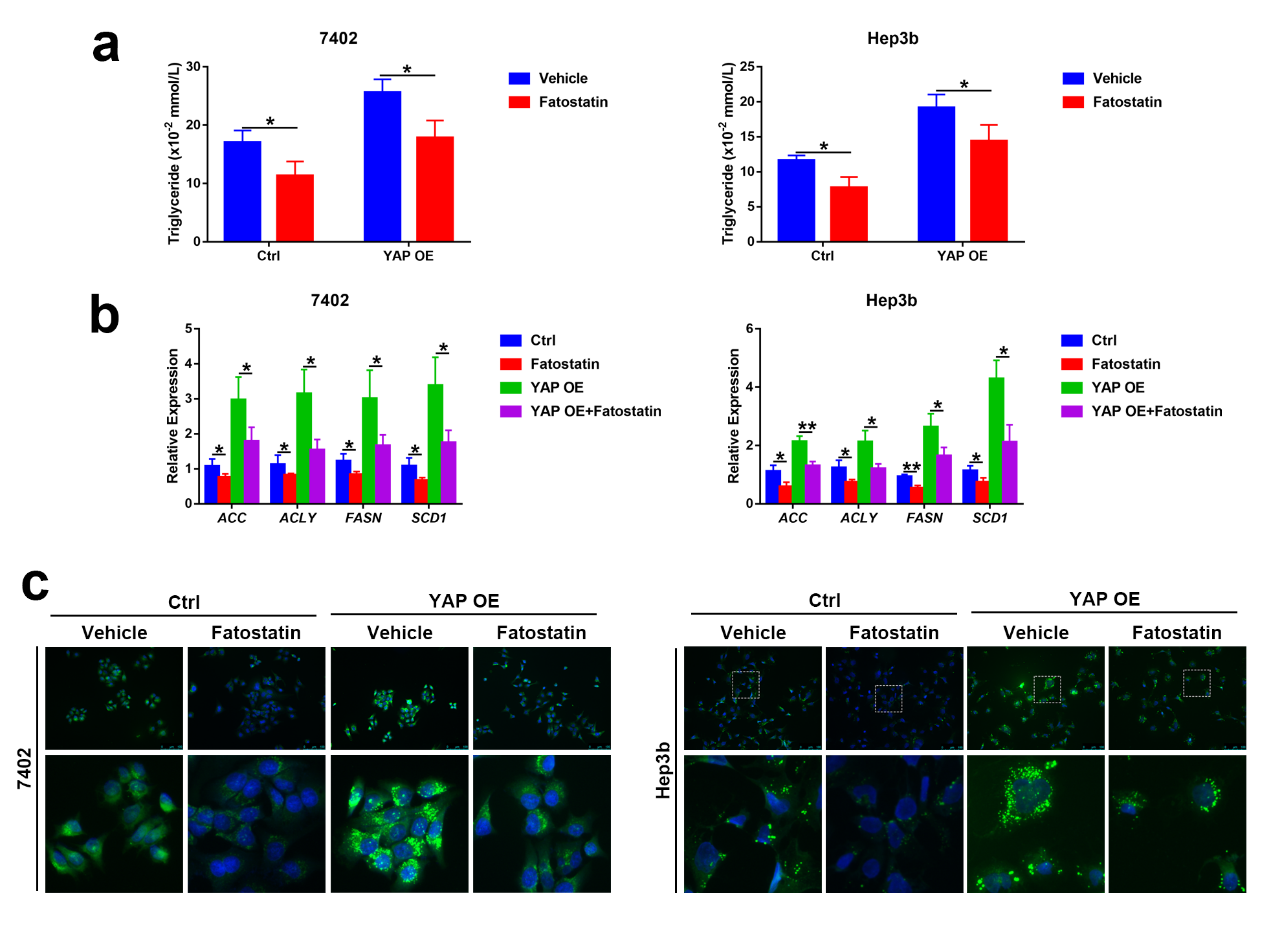


**Figure S5.** YAP regulates lipogenesis in the presence of SREBP1. (a) Cellular TG levels in YAP OE cells treated with SREBP1 inhibitor, Fatostatin. (b) The mRNA levels of lipogenic enzymes in cells treated with Fatostatin (n=3). (c) The content of neutral lipids in cells treated with Fatostatin. (*p<0.05, **p<0.01).
