# Supplementary material for: The hypoxia conditioned mesenchymal stem cells promote hepatocellular carcinoma progression through YAP mediated lipogenesis reprogramming
Source: J Exp Clin Cancer Res. 2019 May 29;38:228. doi: 10.1186/s13046-019-1219-7 (PMC6540399; doi:10.1186/s13046-019-1219-7)
Supplement: Supplementary file 8 — Figure S7. PGE2 activates YAP via EP4 to promote cell proliferation. (a) the mRNA levels of EP1-EP4 in cells treated with PGE2. (b) Expression of EP4 and CREB in cells treated with PGE2. (c) Expression of EP4, CREB and YAP in EP4 knockdown cells treated with PGE2. (d) Quantification of Edu positive cells in EP4 knockdown cells treated with PGE2. (e) The mRNA levels of YAP and its target genes in EP4 knockdown cells treated with PGE2. (*p < 0.05, **p < 0.01, ***p < 0.001). (DOCX 432 kb) [file 13046_2019_1219_MOESM8_ESM.docx]

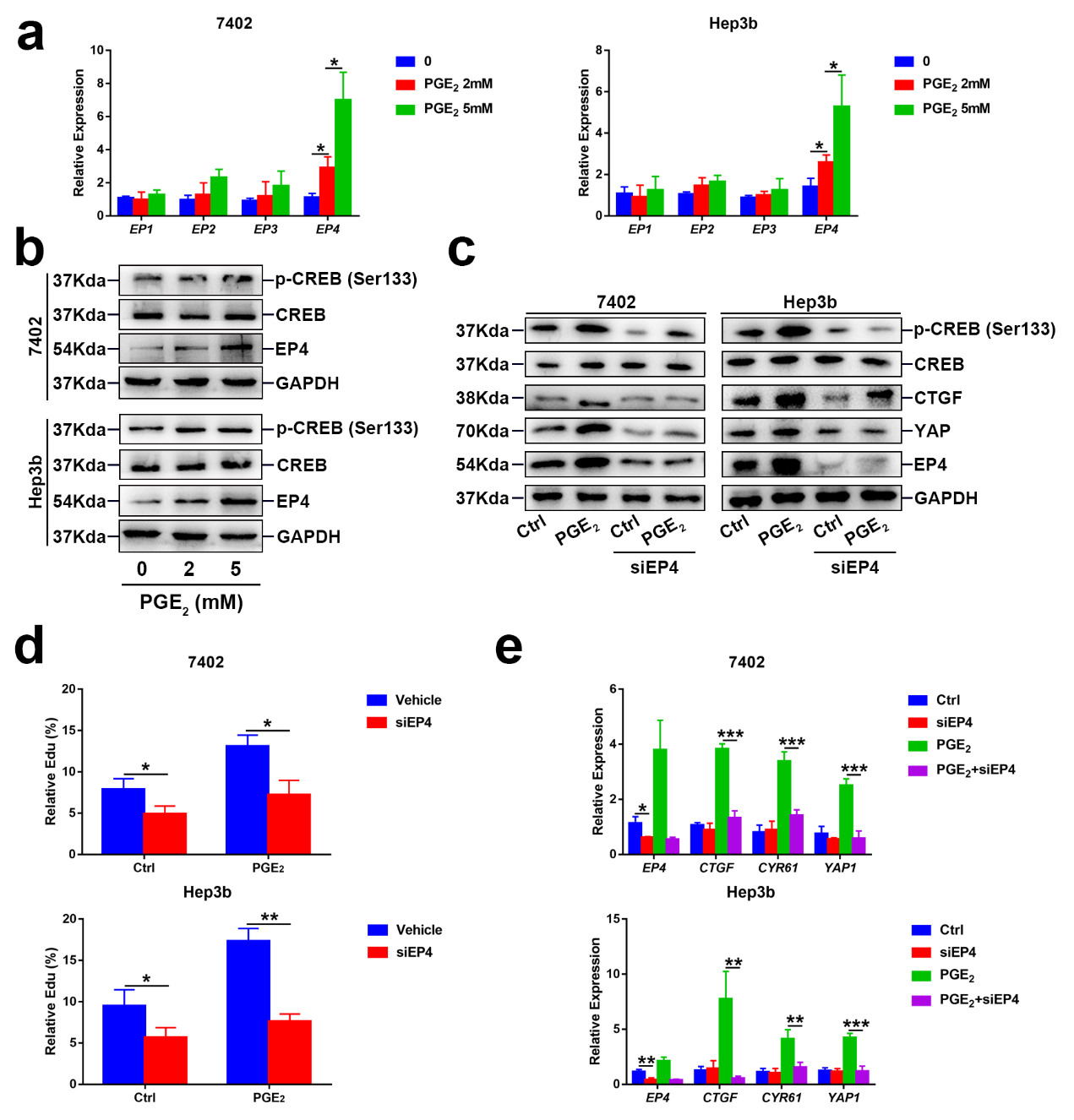


**Figure S7.** PGE_2_ activates YAP via EP4 to promote cell proliferation. (a) the mRNA levels of *EP1*-*EP4* in cells treated with PGE_2_. (b) Expression of EP4 and CREB in cells treated with PGE_2_. (c) Expression of EP4, CREB and YAP in EP4 knockdown cells treated with PGE_2_. (d) Quantification of Edu positive cells in EP4 knockdown cells treated with PGE_2_. (e) The mRNA levels of *YAP* and its target genes in EP4 knockdown cells treated with PGE_2_. (*p<0.05, **p<0.01, ***p<0.001).
