# Supplementary material for: The hypoxia conditioned mesenchymal stem cells promote hepatocellular carcinoma progression through YAP mediated lipogenesis reprogramming
Source: J Exp Clin Cancer Res. 2019 May 29;38:228. doi: 10.1186/s13046-019-1219-7 (PMC6540399; doi:10.1186/s13046-019-1219-7)
Supplement: Supplementary file 9 — Figure S8. The role of GW627368X (EP4 inhibitor, EP4i) on hypo-MSC mediated cell proliferation and lipogenesis. (a) Quantification of Edu positive cells in cells treatment of EP4i under indicated conditions. (b) Expression of YAP in cells treatment of EP4i under indicated conditions. (c) The mRNA levels of YAP and its target genes in cells treatment of EP4i under indicated conditions. (d) Expression of AKT, mTOR and SREBP1 in cells treatment of EP4i under indicated conditions. (e) Cellular TG levels in cells treatment of EP4i under indicated conditions. (f) The content of neutral lipids in cells treatment of EP4i under indicated conditions. EP4i: EP4 inhibitor. (*p < 0.05, **p < 0.01). (DOCX 1210 kb) [file 13046_2019_1219_MOESM9_ESM.docx]

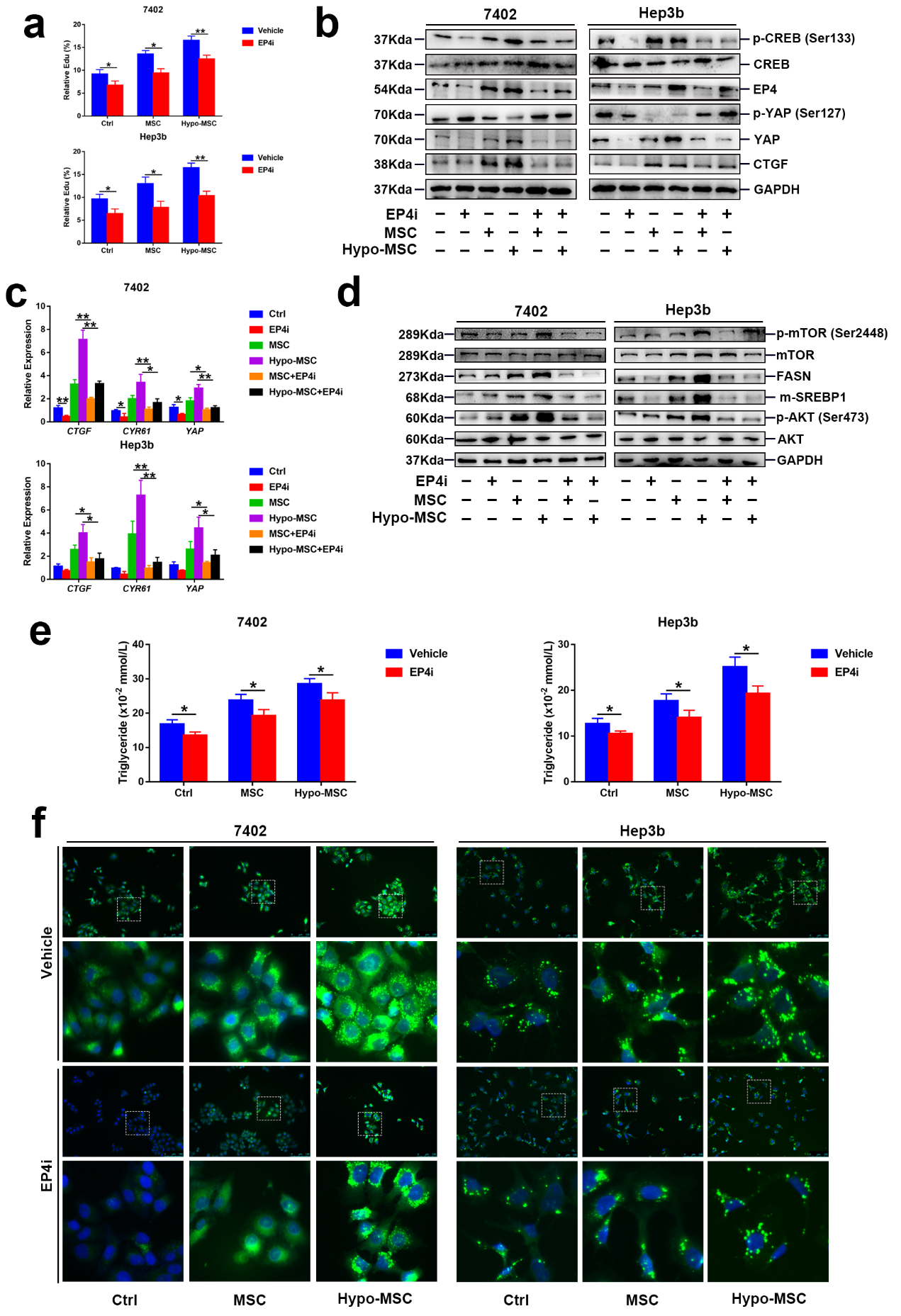


**Figure S8.** The role of GW627368X (EP4 inhibitor, EP4i) on hypo-MSC mediated cell proliferation and lipogenesis. (a) Quantification of Edu positive cells in cells treatment of EP4i under indicated conditions. (b) Expression of YAP in cells treatment of EP4i under indicated conditions. (c) The mRNA levels of *YAP* and its target genes in cells treatment of EP4i under indicated conditions. (d) Expression of AKT, mTOR and SREBP1 in cells treatment of EP4i under indicated conditions. (e) Cellular TG levels in cells treatment of EP4i under indicated conditions. (f) The content of neutral lipids in cells treatment of EP4i under indicated conditions. EP4i: EP4 inhibitor. (*p<0.05, **p<0.01).
